# Pain intensity and psychological distress show different associations with interference and lack of life control: A clinical registry-based cohort study of >40,000 chronic pain patients from SQRP

**DOI:** 10.3389/fpain.2023.1093002

**Published:** 2023-03-02

**Authors:** Björn Gerdle, Elena Dragioti, Marcelo Rivano Fischer, Åsa Ringqvist

**Affiliations:** ^1^ Pain and Rehabilitation Centre, Department of Health, Medicine and Caring Sciences, Linköping University, Linköping, Sweden; ^2^ Department of Neurosurgery and Pain Rehabilitation, Skåne University Hospital, Lund, Sweden; ^3^ Department of Health Sciences, Faculty of Medicine, Lund University, Lund, Sweden

**Keywords:** anxiety, chronic pain, depression, distress, interference, life control, pain intensity, social support

## Abstract

**Background:**

Both chronic pain and depressive and/or anxiety symptoms are associated with negative impacts on daily living, including interference and lack of life control. However, little is known about how pain and psychological distress affect these impacts.

**Aim:**

The first aim was to assess how pain intensity, psychological distress, and social support interact with interference and lack of life control. A second aim was to investigate whether the strength of these relationships is moderated by the presence or absence of depression and/or anxiety.

**Subjects and methods:**

Patient-Reported Outcome Measures (PROMs), which are available in the Swedish Quality Registry for Pain Rehabilitation (SQRP), were retrieved for patients with chronic pain (*N* = 40,184). A theoretical model with the constructs/latent variables pain intensity, psychological distress, interference, lack of life control, and social support was proposed and analyzed using Partial Least Squares Structural Equation Modelling (PLS-SEM). Indicators for these constructs were identified from the PROMs of the SQRP. Two models of the total cohort, which differed with respect to the causal relationship between pain intensity and psychological distress, were investigated. The moderating effects of anxiety and/or depression were also analyzed.

**Results:**

Relatively low correlation and explanatory power (*R*^2 ^= 0.16) were found for the pain intensity-psychological distress relationship. Pain intensity had a stronger effect on interference than on lack of life control. The reverse was found for psychological distress – i.e., psychological distress seemed to have a higher negative influence on function than on interference. The underlying assumption of the causal relationship between pain intensity and psychological distress determined how strong pain intensity and psychological distress influenced interference and lack of life control. Social support showed very similar absolute significant correlations with interference and lack of life control. Interference and lack of life control showed relatively weak associations. The psychological distress level was a moderating factor for several of the paths investigated.

**Discussion and conclusion:**

A clinical treatment consequence of the low correlation between pain intensity and psychological distress may be that clinically treating one may not reduce the effect of the other. The relative importance of pain intensity and psychological distress on interference and lack of life control depends on the underlying assumption concerning the pain intensity-psychological distress relationship. Interference and lack of life control showed relatively weak associations, underscoring the need to clinically assess them separately. Social support influenced both impact constructs investigated. The cohort display heterogeneity and thus presence of definite signs of anxiety and/or depression or not was a moderating factor for several of the associations (paths) investigated. The results are important both for the assessments and the design of treatments for patients with chronic pain.

## 1. Introduction

Chronic pain conditions are associated with increased risks for intense and disturbing pain, psychological distress, disability, poor health, and low quality of life (1–6). Pain is a complex interaction of biological, psychological, social, and contextual factors (7–9). Hence, modern clinical practice uses a biopsychosocial framework when assessing patients and designing and performing interventions (10, 11).

Depression and anxiety are common co-morbidities in chronic pain. Data from the Swedish Quality Registry for Pain Rehabilitation (SQRP) covering specialist care for chronic pain patients indicated that 39.5% of the cohort fulfilled the criteria for a highly probable anxiety case according to Hospital Anxiety and Depression Scale (HADS); the corresponding figure for depressive symptoms was 35.2% (12). Some studies report higher prevalence for depression, which can be due to methods applied and societal factors (13, 14). Complex bidirectional relationships between pain and mood exist according to cross-sectional and longitudinal studies, but these and other studies posit different theories to explain these relationships (13, 15–23). According to a large SQRP study (*N* > 35,000 chronic pain patients), the levels of pain intensity and mood aspects were significantly correlated but with relatively low explained variances (*R*^2^) (pain intensity variables vs. depressive symptoms of HAD: *R*^2 ^= 0.07–0.11; pain intensity vs. anxiety symptoms: *R*^2 ^= 0.07–0.09) (12). A network analysis of another SQRP cohort (*N* = 2,241) confirmed weak correlations between pain intensity and the two mood variables (24).

Both chronic pain and depressive and anxiety symptoms are associated with different negative impacts on daily living and functioning, treatment responses, and health costs (15, 25–34). Thus, pain and psychological distress are associated with interference in daily life and perceived lack of life control (11, 35). For example, a mix of variables (pain intensity, emotion variables, fear, etc.) influence life impact variables such as life control and interference as previously reported from SQRP (35–38). Pain intensity was somewhat more important for interference than depressive symptoms, whereas life control was more strongly associated with symptoms of anxiety and depression (38). However, only 43%–53% of the variations in these two life impact variables were accounted for. Thus, there is an incomplete understanding of what variables determine these aspects. Also, in the network study, pain intensity, depression, and anxiety showed strong associations with pain interference (24). However, for life control aspects, anxiety and depression had stronger relationships than pain intensity (24). Differentiated associations for pain intensity and mood variables in relation to impact aspects have also been reported in tension-type headache (39).

In addition to traditional outcomes such as pain, psychological distress, interference and function, also social outcomes have been advocated as important (40). Social support is defined as the resources received and perceived as being available from others in one's social network (41). Social support can help patients cope with and distract from negative life events and manage pain and its impacts (41–43).

The holistic biopsychosocial model considers complex relationships between the different facets of living with chronic pain. For example, pain intensity may act both *via* direct and indirect (mediating) effects on aspects of life impact. There is a knowledge gap of the relative importance of pain intensity and psychological distress for aspects of life impact such as interference and life control in real life practise settings. Presence of moderating variables indicate cohort heterogeneity. The combination of both pain and depression is associated with poorer treatment outcomes than either condition alone (44, 45), which may indicate that psychological distress is a moderating factor.

**Figure 1:**
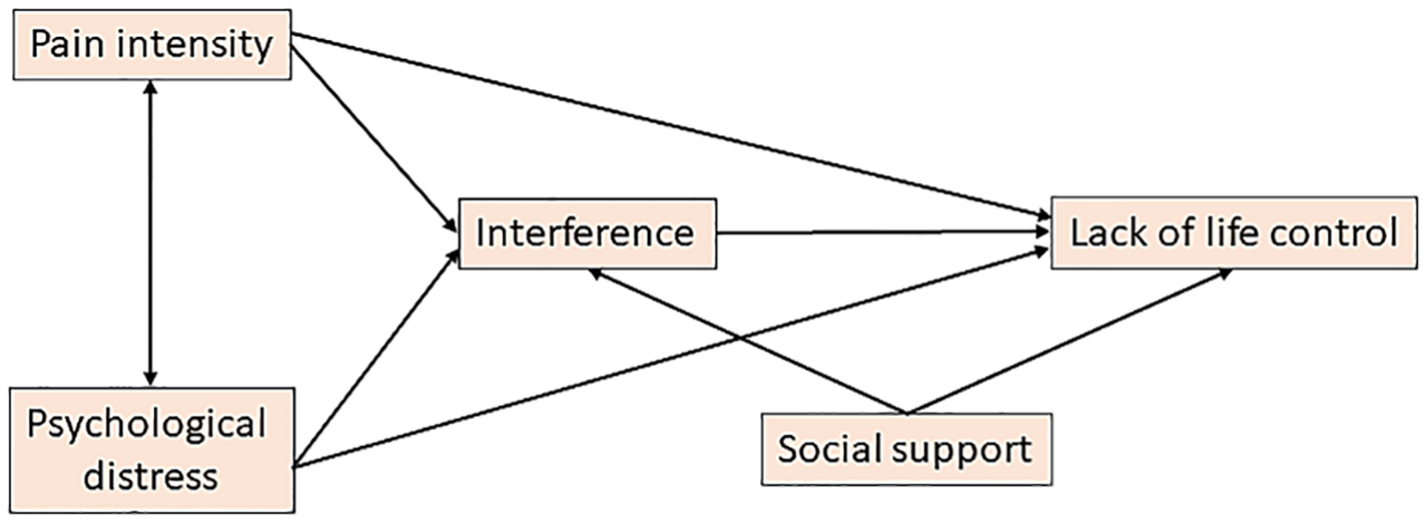
The theoretical model. Constructs/latent variables are shown together with the paths including directions. For the pain intensity-psychological distress relationship, two options (models) are explored (here indicated by a line with an arrowhead at either end).

Partial Least Squares Structural Equation Modelling (PLS-SEM) is a non-parametric method that can be applied to model and estimate complex relationships (paths) among multiple dependent and independent variables (46). Rather than using only one variable for indicating a certain aspect, PLS-SEM is based on latent variables/constructs, which generally are covered by several variables (indicators). Therefore, the construct (e.g., pain intensity) will be more accurately measured (i.e., reduced measurement error) (46). PLS-SEM is always based on a theoretical model – i.e., a complex hypothesis. The relationships between the constructs^1^ in the proposed theoretical model are shown in Figure 1. In agreement with the referred literature, we expected that Pain intensity and Psychological distress were intercorrelated and that both components in turn affect Interference and Lack of life control (47, 48). Interference is the disruption of daily life activities directly due to chronic pain, which, in the literature has been related to a low level of physical function (11). To the best of our knowledge, there is no well-established broader definition of Lack of control. Hence, in this study we define Lack of life control as the impaired ability to control daily life situations, including social life activities. Moreover, we assume that Interference has a positive association with Lack of life control (i.e., prominent interference impairs life control, including social life activities). Furthermore, as a robust social network has a “pain-buffering” effect (49), we hypothesised that Social support affects both the constructs of Interference and Lack of life control.

Earlier studies from this cohort (not imputed data) found that age, sex, education level, country of birth, pain duration, and spatial extent of pain on the body had low correlations with variables indicating our proposed constructs. Therefore, we primarily did not involve them in the theoretical model.

This large clinical registry-based cohort study has two aims. First, using advanced path analysis, we assess how Pain intensity, Psychological distress, and Social support interact directly and indirectly with Interference and Lack of life control. Second, we investigate whether the strength of these relationships is moderated by the presence of definite signs of depression and/or anxiety.

## 2. Materials and methods

### 2.1. Subjects and the Swedish quality registry for pain rehabilitation (SQRP)

The SQRP registers Patient-Reported Outcome Measures (PROMs) data from a majority of the Swedish specialist chronic pain departments (35). This study investigates adult patients (i.e., ≥18 years) with chronic (≥3 months) non-malignant pain registered in the SQRP between 2008 and 2016. Most of the patients were referred by primary care, but some were referred by rheumatology and orthopaedic specialist departments. Patients enrolled in SQRP have complex pain conditions that often include comorbidities (e.g., depression and anxiety), insufficient coping strategies, long sick leave, low social participation, and/or unresponsiveness to monomodal treatments (e.g., pharmacological, and physiotherapeutic interventions). As SQRP is a clinical registry of patients with chronic pain conditions, the SQRP does not have strict inclusion and exclusion criteria. General inclusion criteria include disabling chronic pain (i.e., chronic pain that results in sick leave or major interference in daily life), 18 years and older, medically fully investigated, and written consent to participate. General exclusion criteria include severe psychiatric morbidity, abuse of alcohol and/or drugs, diseases that do not allow physical exercise, and specific pain conditions with other treatment options available (i.e., conditions associated with “red flags”).

### 2.2. Ethics

The study was conducted in accordance with the Helsinki Declaration. It was approved by the Ethical Review Board in Linköping (Dnr: 2015/108-31). All the participants received written information about the study and gave their written consent.

### 2.3. Patient-Reported outcome measures (PROMs)

The PROMs capture a patient's background including demographic aspects, pain intensity, pain-related cognitions, and psychological distress symptoms as well as activity/participation aspects and health-related quality of life variables. PROMs are completed by patients on up to three occasions: before the first visit (baseline assessment), immediately after treatment for those who participated in interdisciplinary pain rehabilitation programs (IPRP; approximately 50%–55% of the patients assessed), and at a 12-month follow-up. In the present study, only baseline data are analysed. In the present baseline study, 19 variables were initially considered for inclusion in the PLS-SEM analyses – i.e., six background variables and 13 of 22 mandatory outcome variables (i.e., variables that all participating clinics/units collect from patients. In addition to this, there are other – non-mandatory – variables that the clinic/unit can include if desired) (Table 1).

**Table 1:** Variables with missing data before imputation – baseline data. Gender, age, and PRI had no missing data. Note that some of these variables were not included in the subsequent analyses (see methods).

	Missing		Valid *N*
	*N*	Percent (%)	
Born outside Europe	1,389	3.4	39,312
University education	1,798	4.4	38,903
Pain duration	5,608	13.8	35,093
NRS-7d	2,090	5.1	38,611
HAD-Anxiety	1,579	3.9	39,122
HAD-Depression	1,561	3.8	39,140
MPI-Pain severity	1,854	4.6	38,847
MPI-Pain interference	2,242	5.5	38,459
MPI-Control	2,019	5.0	38,682
MPI-Distress	1,984	4.9	38,717
MPI-Social Support	2,119	5.2	38,582
MPI-General Activity Index*	2,038	5.0	38,663
EQ5d-index*	3,156	7.8	37,545
EQ-VAS*	3,864	9.5	36,837
sf36-physical function	2,530	6.2	38,171
sf36-role physical	3,310	8.1	37,391
sf36-bodily pain	2,505	6.2	38,196
sf36-general health*	3,034	7.5	37,667
sf36-vitality*	2,627	6.5	38,074
sf36-social function	2,524	6.2	38,177
sf36-role emotional	3,843	9.4	36,858
sf36-mental health	2,672	6.6	38,029

NRS-7d, Pain intensity according to a numeric rating scale; HAD, The Hospital Anxiety and Depression Scale; MPI, Multidimensional Pain Inventory; EQ, The European Quality of Life instrument; EQ5d, EQ Index based on 5 items; EQ-VAS, EQ health variable; sf36, The Short Form Health Survey.

* Not included in the present study (see methods).

#### 2.3.1. Background variables

The following background variables have been described in detail elsewhere (50–53):

• age (years)
• sex
• education level (university vs. no university)
• country of birth (born in vs. outside of Europe)
• pain duration (days)
• spatial extent of pain [Pain Region Index (PRI) – i.e., painful areas marked on 36 predefined anatomical regions] (12, 53)

#### 2.3.2. Mandatory outcome variables in SQRP

The SQRP includes 22 mandatory outcome variables registered on up to three occasions. These variables are consistent with the outcome domains of the Initiative on Methods, Measurement, and Pain Assessment in Clinical Trials (IMMPACT) (11, 54) and the Validation and Application of a patient-relevant core set of outcome domains to assess multimodal PAIN therapy (VAPAIN) (55) initiatives. The mandatory variables, including psychometric properties, have been detailed elsewhere (50–53):

• Mean pain intensity over the past seven days is measured using an 11-point numerical rating scale (0 = no pain to 10 = worst possible pain; NRS-7d).
• The Hospital Anxiety and Depression Scale (HAD) captures signs of depression (HAD-D) and anxiety (HAD-A) (56, 57). Levels ≥11 (possible range: 0–21 for each subscale) indicates a definite case for anxiety or depression. In the multivariate analyses, the two scales (HAD-tot) were summed according to a previous large psychometric study (58).
• The Swedish version of the Multidimensional Pain Inventory (MPI) describes the multidimensional nature of chronic pain. Part one has five subscales: pain severity (MPI-Pain severity), interference (MPI-Pain interference), life control (MPI-Control), psychological distress (MPI-Distress), and perceived social support (MPI-Social Support). MPI-Pain Severity concerns current pain intensity and average pain intensity over the previous seven days. MPI-Pain Interference is based on 11 items covering disturbances due to pain in daily activities, work capacity, leisure activities, general reductions in activities, and the ability to socialize and have relations with family and friends. MPI-Control is based on four items concerning the ability to manage daily life problems, pain, and stressful situations. MPI-Distress is based on three items – mood, irritation, and anxiety in the previous seven days. MPI-Social Support is based on two items – support and help from family or relatives and consideration from family or relatives when having pain. In part two, patients report how they perceive significant others' responses to pain or suffering expressed by the patient in three subscales. The second part was not used in the present study due to high proportions of missing data. Part three measures participation in various activities (i.e., household chores, outdoor work, activities away from home, and being together with family and friends), which are combined into a General Activity Index in the Swedish version (59). The index was not included in the present study due to lack of construct validity (60).
• The European Quality of Life instrument (EQ-5D) measures generic Health-Related Quality of Life (61–63). EQ-5D was not included in the multivariate analyses of the present study.
• The Short Form Health Survey (sf36) assesses eight aspects/dimensions (scored from 0 to 100, with higher scores indicating a better perception) of generic Health-Related Quality of Life (64): (1) physical functioning is a 10-item measure of physical limitation/ability in a range of activities from self-care to exercise (sf36-physical function); (2) role limitations are related to physical functioning including work and daily activities based on four items (sf36-role physical); (3) bodily pain is based on two items measuring pain intensity and the extent pain interferes with daily activities (sf36-bodily pain); (4) general health is a five-item scale concerning self-related health (sf36-general health); (5) the vitality scale is based on four items concerning vitality, energy level, and fatigue with the intention to measure subjective well-being (sf36-vitality); (6) social functioning is based on two items covering the impact of physical and mental health on social functioning (sf36-social function); (7) role limitations due to emotional problems is based on three items (sf36-role emotional); and (8) mental health scale has five items measuring anxiety, depression, loss of behavioural/emotional control, and psychological well-being (sf36-mental health) (65). In the present study, sf36-general health and sf36-vitality were not included in the multivariate analyses as they measure global aspects of health and wellbeing, which are not the focus of this study.

#### 2.3.3. Identification of indicators for the constructs/latent variables

The constructs according to our theoretical model/hypotheses are shown in Figure 1. Both MPI and sf36 are comprehensive instruments and composed by several subscales. Clinical reasoning as well as descriptions in the literature of the different subscales were applied when determining the usefulness of the above selected SQRP variables as indicators in relation to the latent variables (Table 2) (59, 65–67). Since all indicators of a certain construct must be positively intercorrelated some of the potential indicators were reversed. We identified three variables that mainly measure pain intensity – NRS-7d, MPI-Pain severity, and sf-36-bodily pain (was reversed). We identified four indicators of psychological distress – HAD-tot, MPI-Distress, sf36-mental health (reversed), and sf36-role emotional (reversed). For Interference, we identified three indicators – MPI-Pain interference, sf36-physical function (reversed), and sf36-role physical (reversed). Although these aspects are interchangeably labelled as Interference and physical functioning (68), we chose the former expression to reflect interference and associated physical function (11). Our construct Interference agrees with other studies that bring these together into one domain, at least in cross-sectional studies (47, 68). Thus, our concept Interference covers pain interference and physical function (11). The Lack of life control construct indicates the inability to control life aspects, including social life activities. MPI-Control (reversed) and sf36-social function (reversed) were identified as indicators. MPI-Social support was used as a single indicator of social support, which mainly reflects support from a spouse and significant others.

**Table 2:** Summary of constructs/latent variables and indicators included in the initial PLS-SEM analysis of the total cohort.

Construct/Latent variable	Indicators
Pain intensity	NRS-7d
	MPI-Pain severity
	Sf36-bodily pain-rev
Psychological distress	HAD-tot
	MPI-Distress
	sf36-mental health-rev
	sf36-role emotional-rev
Lack of life control	MPI-Control-rev
	sf36-social function-rev
Interference	MPI-Pain interference
	sf36-physical function-rev
	sf36-role physical-rev
Social support	MPI-Social support

-rev, the variable was revised to indicate a troublesome situation.

NRS-7d, Pain intensity according to a numeric rating scale; HAD, The Hospital Anxiety and Depression Scale; HAD-tot, sum of the two subscales of HAD; MPI, Multidimensional Pain Inventory; sf36, The Short Form Health Survey.

As mentioned above, earlier analyses have found that age, sex, education level, country of birth, pain duration, and spatial extent of pain (i.e., the PRI) have low correlations with variables indicating our constructs. At first, we did not use them in the theoretical model. However, we eventually decided to include them in the theoretical model if our previous results were not confirmed using imputed data for the cohort.

### 2.4. Statistics

We used three statistical packages: IBM SPSS Statistics (version 28.0; IBM Corporation, Route 100 Somers, New York, United States); SIMCA-P + (version 17.0; Sartorius Stedim Biotech, Umeå, Sweden); and Smart-PLS version 3 (Ringle, C. M., Wende, S., and Becker, J.-M. 2015. “SmartPLS 3” Boenningstedt: SmartPLS GmbH, http://www.smartpls.com). In the text and tables, the mean value ± one standard deviation (±1 SD) of continuous variables and percentages (%) for categorical variables are reported. To compare groups, Student's *t*-test for independent samples and Chi squared tests were applied. In large samples, small differences may be significant and effects size can be used to evaluate the clinical importance of the significant differences. Effect sizes (ES; Cohen's *d*) for within group analyses were computed and Hedges' *g* – a measure of effect size weighted by the relative size of each sample – was used for between group ESs. The absolute effect size was considered clinically insignificant for <0.20, small for 0.20–0.49, moderate for 0.50–0.79, and large for ≥0.80 (69).

The retrieved SQRP data included missing data (Table 1). Our analysis of missingness mechanisms indicated that data were not missing completely at random (MCAR) (i.e., Little's Test of Missing Completely at Random was not significant) and therefore data were missing either at random (MAR) or not missing at random (NMAR) (70). One choice may have been to use listwise deletion, but, multiple imputation gives less biased results than listwise deletion (70). The multiple imputation option in SPSS was used for the imputation of our variables (Random generator: Mersenne Twister with a fixed starting point for the active generator initialization; all variables were included in five imputations, which were pooled). The imputed data are shown in Table 3.

**Table 3:** Mean and SD for the imputed data for the total cohort (*N* = 40,186 for all variables) (left). On the right is shown the two subgroups (i.e., Low distress (*N* = 20,986) and High distress (*N* = 19,288). Furthest to the right are shown the results of the comparisons between the two subgroups (*P*-value and effect size ES (Hedges' *g*) for the quantitative variables.

Proportion	All (%)		Low distress (%)	subgroup	High Distress (%)	subgroup	Statistics *P*	ES
Men	27.9		27.7		28.0		0.495	NA
Born outside Europe	15.1		9.9		20.7		<0.001	NA
University education	25.0		28.0		21.7		<0.001	NA
	Mean	SD	Mean	SD	Mean	SD	*P*	ES
Age	43.2	11.4	43.7	11.7	42.6	11.0	<0.001	0.10
PRI	13.9	8.9	12.2	8.5	15.8	9.0	<0.001	−0.40
Pain-duration	3088	3097	3034	3082	3147	3112	<0.001	−0.04
NRS-7d	6.8	2.0	6.3	2.1	7.3	1.9	<0.001	−0.49
HAD-tot	17.6	8.9	10.6	4.8	25.1	5.8	<0.001	−2.72
MPI-Pain severity	4.3	1.2	4.0	1.3	4.7	1.0	<0.001	−0.59
MPI-Pain interference	4.2	1.3	3.8	1.3	4.7	1.1	<0.001	−0.77
MPI-Control	2.6	1.2	3.0	1.2	2.1	1.1	<0.001	0.84
MPI-Distress	3.4	1.4	2.6	1.2	4.2	1.1	<0.001	−1.36
MPI-Social support	4.0	1.5	4.0	1.5	4.0	1.5	<0.001	−0.03
sf36-physical function	48.7	23.2	52.1	23.3	45.0	22.4	<0.001	0.31
sf36-role physical	14.1	25.0	17.9	27.7	10.0	20.8	<0.001	0.33
sf36-bodily pain	22.8	15.0	25.8	15.0	19.5	14.3	<0.001	0.43
sf36-social function	44.7	26.3	54.5	26.2	34.2	21.9	<0.001	0.84
sf36-role emotional	41.2	41.4	58.0	41.1	22.9	33.3	<0.001	0.93
sf36-mental health	51.5	23.7	63.7	21.5	38.3	18.3	<0.001	1.27

PRI, Pain Region Index, NRS-7d, Pain intensity according to a numeric rating scale; HAD, The Hospital Anxiety and Depression Scale; HAD-tot, sum of the two subscales of HAD; MPI, Multidimensional Pain Inventory; sf36, The Short Form Health Survey.

Previous studies have discussed the necessity of using advanced multivariate analyses (MVDA) when accounting for system-wide aspects and multicollinearity problems (53, 71). Advanced Principal Component Analysis (PCA), which is available in SIMCA-P+, can be used to determine multivariate outliers and multivariate correlation patterns. Outliers were identified using score plots in combination with Hotelling's *T*^2^ and distance to model in X-space. PCA can conceptually be viewed as a kind of multivariate correlation analysis that extracts and displays systematic variation in the data matrix. To identify nontrivial components (*p*), we used a cross validation technique. Variables loading on the same component *p* are correlated; variables with high loadings but opposing signs are negatively correlated. Variables with high absolute loadings were considered significant. The obtained components are not correlated but are arranged in decreasing order with respect to explained variation. *R*^2^ describes the goodness of fit – the fraction of sum of squares of all the variables explained by a principal component (72). Q^2^ describes the goodness of prediction – the fraction of the total variation of the variables that can be predicted using principal component cross validation methods (72).

Orthogonal Partial Least Square Regressions (OPLS) was used for the multivariate regression analyses of pain intensity and psychological distress variables. The variable influence on projection (VIP) indicates the relevance of each *X*-variable pooled over all dimensions and *Y*-variables – the group of variables that best explains *Y* (72). A variable was considered significant when VIP > 1.0 (or VIPpred if > one component) and had a 95% jack-knife uncertainty confidence interval non-equal to zero. *P*(corr) was used to note the direction of the relationship (positive or negative) – i.e., the loading of each variable was scaled as a correlation coefficient and therefore standardised the range from −1 to +1 (71). Thus, a variable/regressor was considered statistically significant when VIP or VIPpred > 1.0. A regression model was obtained, including one or several components (the first is always the predictive component), if certain predefined criteria were fulfilled. The validity of the model was estimated using cross validation. Hence, for each regression, we report *R*^2^, *Q*^2^, and the *P*-value of a cross-validated analysis of variance (CV-ANOVA).

SMART-PLS version 3 (Ringle, C. M., Wende, S., and Becker, J.-M. 2015. “SmartPLS 3.” Boenningstedt: SmartPLS GmbH, http://www.smartpls.com) was used for the Partial Least Squares Structural Equation Modelling (PLS-SEM). This non-parametric method enables researchers to simultaneously model and estimate complex relationships among multiple dependent and independent variables. The method has a causal-predictive approach to SEM, which intends to explain the variance of the dependent variables; for details of this method, see (46). The recommendations by Hair Jr et al. were followed when performing the PLS-SEM analyses (46). This method is based on constructs (latent variables) that are generally associated with several indicators. The indicators of a certain component must have the same direction – i.e., they cannot be negatively intercorrelated. The measurement model (outer model) displays the relationships between the constructs and its indicator variables. In the present study, a reflective relationship was assumed for all indicator-construct relationships (not relevant for single indicator constructs). Exogenous latent variables are constructs that explain other constructs – i.e., endogenous latent variables (46). The structural model displays the relationships (paths) between the constructs. *Indicator reliability* was determined using the outer loadings (possible range: −1–1), and it is generally required that the absolute loadings are >0.708. It is recommended that indicators with absolute loadings ≤0.40 be omitted, and indicators with absolute loadings between 0.40 and 0.708 be omitted only if this result increased internal consistency or convergent validity. *Internal consistency reliability* was determined using exact (consistent) reliability coefficient (*ρ*_A_; possible range: 0–1), and >0.50 was required. *Convergent validity* was determined using Average Variance Extracted (AVE; possible range: 0–1), and >0.50 was required. Heterotrait-monotrait ratio (HTMT) was used as an indicator of *discriminant validity*, and we required values <0.90, preferably <0.85.

To evaluate the structural/inner model, presence of *collinearity* was checked using variance inflation factor (VIF), and values < 5 were acceptable, preferably <3 (46). *Path coefficients* (i.e., standardized regression coefficients displaying direct effects) were determined, including mean ± SD, *t*-values, *p*-values, and 95% confidence intervals (95% CI). To determine path coefficients and total effects, indirect effects and specific indirect effects, we performed bootstrapping (options: 10,000 samples, complete bootstrapping, percentile boot strap, two-tailed, *p* = 0.05). *Explanatory power* was determined from coefficient of determination (*R*^2^, possible range: 0–1) as well as *f*^2^ effect size. The latter expresses the change in *R*^2^ when a specific predecessor construct is omitted from the model (<0.02 = no measurable effect; 0.02–0.14 = small effect; 0.15–0.34 = medium effect; and ≥0.35 large effect). Bootstrapping was performed to obtain *t*-values, *p*-values, and 95% CI of these coefficients. In this explorative study, *predictive power* was not in focus. However, using the PLS_predictive_ option (10 folds and 10 repetitions), we determined *Q*^2^_predictive_ values. Values > 0 indicated that the model had predictive relevance. The greater value of *Q*^2^, the greater predictability of the model: < 0.02 = no predictive relevance, 0.02–0.14 = small relevance, 0.15–0.34 = relevance, and ≥0.35 = large predictive relevance (73).

## 3. Results

### 3.1. Descriptive data for the total cohort

The imputed data are shown in Table 3. Most patients were women, a large majority were born in Europe, and one-fourth had a university education. Patients had on average a relatively high pain intensity and reported high levels of psychological distress and negative effects upon interference, physical and social function, and life control. The prevalence of definite cases for anxiety and depression according to HAD were 38.0% and 33.9%. Hence, this clinical pattern is consistent with non-imputed data for this cohort (12, 53).

### 3.2. PCA including check for multivariate outliers

**Figure 2:**
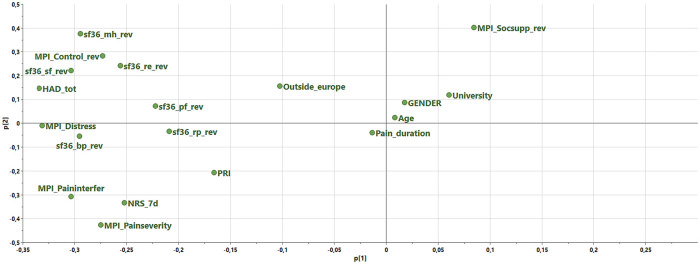
Loadings of a principal component analysis (PCA) of the included variables for the total cohort (N = 40,184). Two components (p1 and p2) are shown. Note that some of the MPI variables and all sf36 variables were revised (indicated with _rev in the variable name) so that high values indicated a troublesome/negative situation. NRS_7d, Pain intensity according to a numeric rating scale; University, University education (binary variable); Outside_Europe, born outside Europe (binary variable), PRI, Pain region Index; HAD, The Hospital Anxiety and Depression Scale; HAD-tot, sum of the two subscales of HAD; MPI, Multidimensional Pain Inventory; MPI_Paininterfer, MPI Pain interference; MPI_Painseverity, MPI Pain severity; MPI_Socsupp, MPI Social support; sf36, The Short Form Health Survey; sf36_pf, sf36-physical function; sf36_rp, sf36-role physical; sf36_bp, sf36-bodily pain; sf36_sf, sf36-social function; sf36_re, sf36-role emotional; sf36_mh, sf36-mental health.

Using a PCA in SIMCA-P+, we found two strong multivariate outliers (MCEID: 12543 and 16266), so we excluded them from the subsequent analysis. Thereafter, a PCA of the included variables (*R*^2^_cummulative _= 0.38, *Q*^2^_cummulative _= 0.25) was conducted (some MPI variables and all sf36 variables were reversed so that high values indicated a troublesome/negative situation) (Figure 2), which identified two significant components. Important variables for the first component (*R*^2 ^= 0.27, *Q*^2 ^= 0.19) were psychological variables (sf36-mental health, HAD-tot, MPI-Distress, and sf36-role emotional) as well as pain intensity variables (sf36-bodily pain, NRS-7d, and MPI-Pain severity). Also, interference (MPI-Pain interference), physical function aspects (sf36-physical function and sf36-role physical), social aspects (sf36-social function), and control (MPI-Control) had importance for this component. The second component (*R*^2 ^= 0.12, *Q*^2 ^= 0.05) mainly reflected social support (MPI-Social support) but also mental health (sf36-mental health) and control aspects (MPI-Control). Several of the sociodemographic variables showed low importance (loaded near zero) – i.e., pain duration, age, gender, university, outside-Europe, and PRI.

### 3.3. OPLS regressions of psychological distress and pain intensity variables

In the next step, the four psychological variables and the three pain intensity variables were regressed (Supplementary Tables S1, S2). As expected, the psychological distress variables and the three pain intensity variables were generally intercorrelated. Pain intensity and psychological distress exhibited different strengths in their relationships to the impacts. Hence, psychological distress aspects were associated with control (MPI-Control), and social function (sf36-social function) in the four regressions and with interference in three of the regressions (MPI-Pain interference). Generally, MPI-Control was a stronger regressor than MPI-Pain interference. In the regressions of the pain intensity variables, pain interference was an important regressor in three regressions, and physical function (sf36-physical function) and social support (MPI-Social support) were important regressors (*X*-variables) in two regressions.

### 3.4. PLS SEM of the total cohort

#### 3.4.1. Selection of variables for PLS-SEM analyses

**Figure 3:**
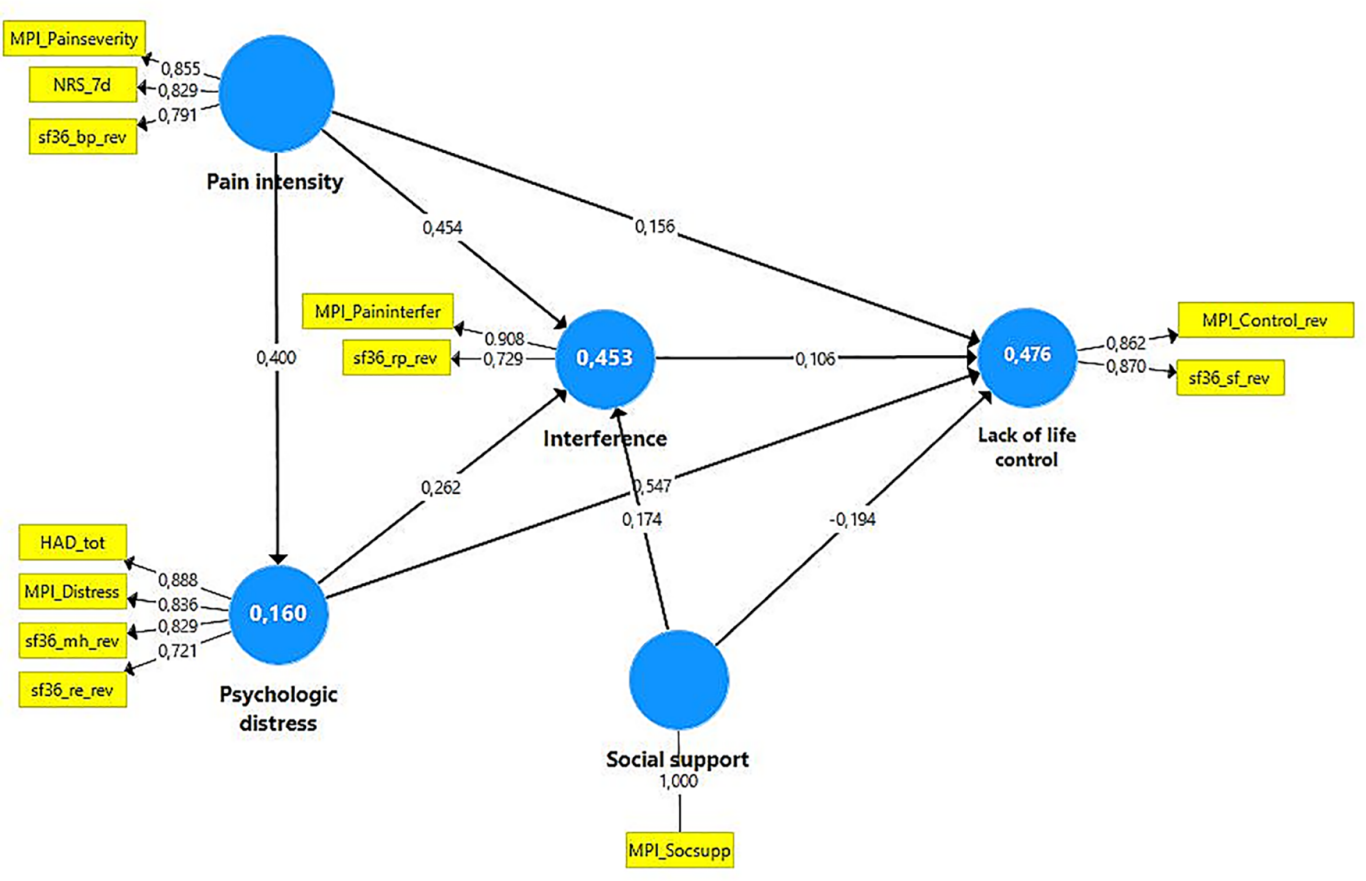
Model 2 for all subjects (*N* = 40,184). That is, if pain intensity affects psychological distress, model 1 is identical including loadings and path coefficients except for the arrow between pain intensity and psychological distress and *R*^2^ for pain intensity then is 0.160. The blue circles show the constructs/latent variables. The yellow boxes show the indicators (reflective) for each construct/latent variable; the figure attached to the thin arrows show the loading. The thicker arrows show the suggested relations between the constructs, and the figure attached shows the path coefficient (direct effect). The explained variance (*R*^2^) is reported within the relevant constructs. Note that some of the MPI variables and all sf36 variables were revised (indicated with _rev in the variable name) so that high values indicated a troublesome/negative situation. NRS_7d, Pain intensity according to a numeric rating scale; HAD, The Hospital Anxiety and Depression Scale; HAD-tot, sum of the two subscales of HAD; MPI, Multidimensional Pain Inventory; MPI_Paininterfer, MPI Pain interference; MPI_Painseverity, MPI Pain severity; MPI_Socsupp, MPI Social support; sf36, The Short Form Health Survey; sf36_rp, sf36-role physical; sf36_bp, sf36-bodily pain; sf36_sf, sf36-social function; sf36_re, sf36-role emotional; sf36_mh, sf36-mental health.

To achieve a parsimonious model in the PLS-SEM analysis, only variables with absolute loadings >0.20 in the PCA were included (Figure 2). In addition, the OPLS regressions indicated that these excluded variables had low importance (Supplementary Tables S1, S2). The included indicators in the subsequent PLS-SEM analyses as well as their constructs/latent variables are listed in Table 2. We assumed that psychological distress directly affected pain intensity (model 1) and then assumed that pain intensity directly affected psychological distress (model 2) (Figure 3).

#### 3.4.2. Evaluation of the reflective measurement (outer) models

Indicator reliability was above the threshold of 0.708 for all indicators except sf36-physical function and sf36-role physical, but these had absolute values well above the level used for elimination (i.e., 0.40). Internal consistency reliability according to the exact (consistent) reliability coefficient was satisfactory for all latent variables as well as convergent validity according to average variance extracted (AVE) – i.e., both >0.50. The discriminant validity was satisfactory for all relevant latent variable combinations except for pain vs. interference according to HTMT. When sf36-physical function was excluded as an indicator of interference, all HTMT values were below 0.90 (i.e., indicating discriminant validity). In addition, the indicator reliability for all indicators was above the threshold of 0.708. Loadings are shown in Figure 3 and the other final characteristics are shown in Supplementary Table S3. The two models were identical with respect to indicator reliability, internal consistency reliability, convergent validity, and discriminant validity (Figure 3 and Supplementary Table S3). To conclude, the final outer model for the total cohort was associated with good indicator reliability, internal consistency reliability, convergent validity, and discriminant validity in both models.

#### 3.4.3. Evaluation of the structural (inner) models

VIF values were below 2 (1.0–1.83) – i.e., model collinearity was not an issue in either of the two models. Thereafter, the path coefficients (direct effects) as well as total and indirect effects were determined (Tables 4A,B). All path coefficients (direct effects), total effects, and indirect effects were significant in both models (<0.001) (Table 4A). Effect sizes (*f*^2^) are shown in Table 5.

**Table 4A:** Path coefficients (direct effects), total and total indirect effects for the two models of the total cohort (*N* = 40,184). In model 1, psychological distress → Pain intensity and in model 2 the opposite (i.e., Pain intensity → Psychological distress). Mean ± SD, *t*-values, *P*-values, and 95% confidence intervals (95% CI) are reported.

	Model 1				Model 2				Sign
	Mean ± SD	*t*	*P*	95% CI	Mean ± SD	*t*	*P*	95% CI	
**Path coefficients (direct effects)**									
Psychologic distress → Pain intensity*	0.400 ± 0.004	90.562	<0.001	0.391–0.408	0.400 ± 0.004	90.185	<0.001	0.391–0.408	NA
Pain intensity → Interference	0.454 ± 0.005	99.721	<0.001	0.445–0.463	0.454 ± 0.005	100.547	<0.001	0.445–0.463	NA
Pain intensity → Lack of life control	0.156 ± 0.005	30.124	<0.001	0.146–0.166	0.156 ± 0.005	29.854	<0.001	0.146–0.166	NA
Psychologic distress → Interference	0.262 ± 0.004	64.117	<0.001	0.254–0.270	0.262 ± 0.004	64.321	<0.001	0.254–0.270	NA
Psychologic distress → Lack of life control	0.547 ± 0.004	138.880	<0.001	0.540–0.555	0.547 ± 0.004	139.366	<0.001	0.540–0.555	NA
Social support → Interference	0.174 ± 0.005	38.448	<0.001	0.165–0.183	0.174 ± 0.004	38.699	<0.001	0.165–0.182	NA
Social support → Lack of life control	−0.194 ± 0.004	45.412	<0.001	−0.202–−0.185	−0.194 ± 0.004	45.709	<0.001	−0.202–­−0.185	NA
Interference → Lack of life control	0.106 ± 0.006	19.080	<0.001	0.095–0.117	0.106 ± 0.006	19.039	<0.001	0.095–0.117	NA
**Total effects**									
Psychologic distress → Pain intensity*	0.400 ± 0.004	90.562	<0.001	0.391–0.408	0.400 ± 0.004	90.185	<0.001	0.391–0.408	N
Pain intensity → Interference	0.454 ± 0.005	99.721	<0.001	0.445–0.463	0.559 ± 0.004	145.294	<0.001	0.552–0.567	Y
Pain intensity → Lack of life control	0.204 ± 0.005	43.028	<0.001	0.195–0.213	0.434 ± 0.005	86.987	<0.001	0.424–0.444	Y
Psychologic distress → Interference	0.444 ± 0.004	115.322	<0.001	0.436–0.451	0.262 ± 0.004	64.321	<0.001	0.254–0.270	Y
Psychologic distress → Lack of life control	0.657 ± 0.003	206.561	<0.001	0.650–0.663	0.575 ± 0.004	156.909	<0.001	0.568–0.582	Y
Social support → Interference	0.174 ± 0.005	38.448	<0.001	0.165–0.183	0.174 ± 0.004	38.699	<0.001	0.165–0.182	N
Social support → Lack of life control	−0.175 ± 0.004	40.107	<0.001	−0.184–−0.167	−0.175 ± 0.004	40.278	<0.001	−0.184–−0.167	N
Interference → Lack of life control	0.106 ± 0.006	19.080	<0.001	0.095–0.117	0.106 ± 0.006	19.039	<0.001	0.095–0.117	N
**Total indirect effects**									
Psychologic distress → Pain intensity*	NA	NA	NA	NA	NA	NA	NA	NA	NA
Pain intensity → Interference	NA	NA	NA	NA	0.105 ± 0.002	52.077	<0.001	0.101–0.109	NA
Pain intensity → Lack of life control	0.048 ± 0.003	18.746	<0.001	0.043–0.053	0.278 ± 0.004	72.346	<0.001	0.270–0.286	Y
Psychologic distress → Interference	0.182 ± 0.003	71.791	<0.001	0.177–0.186	NA	NA	NA	NA	NA
Psychologic distress → Lack of life control	0.109 ± 0.003	40.160	<0.001	0.104–0.115	0.028 ± 0.002	17.869	<0.001	0.025–0.031	Y
Social support → Lack of life control	0.018 ± 0.001	17.899	<0.001	0.016–0.020	0.018 ± 0.001	17.92	<0.001	0.016–0.020	N
Interference → Lack of life control									

N, not significant (i.e., overlapping 95% CI); NA, not applicable; Y, significant (i.e., non-overlapping 95% CI for the two subgroups).

* In model 2 reversed – i.e., Pain intensity → Psychological distress.

**Table 4B:** Specific indirect effects for the two models of the total cohort (*N* = 40,184). In model 1 psychological distress → Pain intensity and in model 2 the opposite (i.e., Pain intensity → Psychological distress). Mean ± SD, *t*-values, *P*-values, and 95% confidence intervals (95% CI) are reported.

	Model 1					Model 2			
	Mean ± SD	*t*	*P*	95% CI		Mean SD	*t*	*P*	95% CI
Pain intensity → Interference → Lack of life control	0.048 ± 0.003	18.746	<0.001	0.043–0.053	Pain intensity → Interference → Lack of life control	0.048 ± 0.003	18.751	<0.001	0.043–0.053
Psychologic distress → Interference → Lack of life control	0.028 ± 0.002	17.858	<0.001	0.025–0.031	Psychologic distress → Interference → Lack of life control	0.028 ± 0.002	17.869	<0.001	0.025–0.031
Psychologic distress → Pain intensity → Lack of life control	0.062 ± 0.002	27.924	<0.001	0.058–0.067	Pain intensity → Psychologic distress → Lack of life control	0.219 ± 0.003	74.709	<0.001	0.213–0.225
Social support → Interference → Lack of life control	0.018 ± 0.001	17.899	<0.001	0.016–0.020	Social support → Interference → Lack of life control	0.018 ± 0.001	17.920	<0.001	0.016–0.020
Psychologic distress → Pain intensity → Interference → Lack of life control	0.019 ± 0.001	18.52	<0.001	0.017–0.021	Pain intensity → Psychologic distress → Interference → Lack of life control	0.011 ± 0.001	17.570	<0.001	0.010–0.012
Psychologic distress → Pain intensity → Interference	0.182 ± 0.003	71.791	<0.001	0.177–0.186	Pain intensity → Psychologic distress → Interference	0.105 ± 0.002	52.077	<0.001	0.101–0.109

**Table 5:** F-square values (*f*^2^) for the total cohort (model 1 and 2) (*N* = 40,184) and for the two subgroups Low distress (*N* = 20,986) and high distress (*N* = 19,288). Mean ± SD, *t*-values, *P*-values, and 95% confidence intervals (95% CI) are reported.

*f* ^2^	Model 1				Model 2			
	Mean ± SD	*t*	*P*	95% CI	Mean ± SD	*t*	*P*	95% CI
Psychologic distress → Pain Intensity M1 or Pain intensity → Psychologic distress M2	0.190 ± 0.005	38.033	<0.001	0.181–0.200	0.190 ± 0.005	38.033	<0.001	0.181–0.200
Pain Intensity → Interference	0.278 ± 0.007	40.041	<0.001	0.264 0.292	0.278 ± 0.007	40.041	<0.001	0.264–0.292
Pain Intensity → Lack of life control	0.027 ± 0.002	14.556	<0.001	0.023–0.031	0.027 ± 0.002	14.556	<0.001	0.023–0.031
Psychologic distress → Interference	0.104 ± 0.003	30.879	<0.001	0.097–0.110	0.104 ± 0.003	30.879	<0.001	0.097–0.110
Psychological distress → Lack of life control	0.428 ± 0.008	53.134	<0.001	0.412–0.443	0.428 ± 0.008	53.134	<0.001	0.412–0.443
Social support → Interference	0.048 ± 0.003	19.062	<0.001	0.043–0.053	0.048 ± 0.003	19.062	<0.001	0.043–0.053
Social support → Lack of life control	0.060 ± 0.003	23.113	<0.001	0.055–0.065	0.060 ± 0.003	23.113	<0.001	0.055–0.065
Interference → Lack of life control	0.012 ± 0.001	9.319	<0.001	0.009–0.014	0.012 ± 0.001	9.319	<0.001	0.009–0.014
***f* ^2^**	**Low distress subgroup**				**High distress subgroup**			
Pain Intensity → Psychological distress	0.05 ± 0.004	12.803	<0.001	0.042–0.057	0.166 ± 0.007	24.885	<0.001	0.153–0.179
Pain Intensity → Interference	0.312 ± 0.009	33.353	<0.001	0.294–0.330	0.253 ± 0.011	22.544	<0.001	0.231–0.275
Pain Intensity → Lack of life control	0.029 ± 0.003	8.968	<0.001	0.022–0.035	0.035 ± 0.003	10.42	<0.001	0.029–0.042
Psychological distress → Interference	0.065 ± 0.004	16.707	<0.001	0.057–0.072	0.039 ± 0.003	12.542	<0.001	0.033–0.046
Psychological distress → Lack of life control	0.252 ± 0.008	29.797	<0.001	0.236–0.269	0.274 ± 0.009	29.834	<0.001	0.256–0.292
Social support → Interference	0.073 ± 0.004	16.282	<0.001	0.064–0.082	0.031 ± 0.003	10.58	<0.001	0.026–0.037
Social support → Lack of life control	0.109 ± 0.005	21.559	<0.001	0.099–0.119	0.023 ± 0.002	10.27	<0.001	0.019–0.027
Interference → Lack of life control	0.011 ± 0.002	6.633	<0.001	0.008–0.014	0.018 ± 0.002	7.361	<0.001	0.014–0.023

M1, model 1; M2, model 2.

##### 3.4.3.1. Direct effects (path coefficients)

The path coefficients were identical in the two models (Table 4A and Figure 3). Psychological distress vs. Pain intensity (model 1) showed a relatively prominent direct path coefficient (and the reverse in model 2) (0.40; medium *f*^2^). Pain intensity had a relatively strong association with Interference (0.45; medium *f*^2^) and a weaker direct association (0.16; small *f*^2^) with Lack of life control. Psychological distress had a stronger direct association with Lack of life control (0.55; large *f*^2^) than with Interference (0.26; small *f*^2^). Interference vs. Lack of life control had a small path coefficient (0.11; no measurable effect). Social support had a positive path coefficient with Interference (0.17; small *f*^2^) and a negative (−0.19; small *f*^2^) with Lack of life control.

##### 3.4.3.2. Total effects

To some extent, total effects differed between the two models (Tables 4A,B). In both models, the total effects for Pain intensity were stronger on Interference than on Lack of life control (Table 4A). These total effects for Pain intensity were stronger in model 2 than in model 1. In both models, Psychological distress showed stronger total effects on Lack of life control than for Interference (Table 4A). These total effects for Psychological distress on Lack of life control and Interference were stronger in model 1 than in model 2.

The total effects of Pain intensity and Psychological distress on Interference were similar in model 1 (0.45 vs. 0.44) (Table 4A). However, prominent differences existed in model 2; corresponding effects were 0.56 and 0.26 (Table 4A). The total effects of Pain intensity and Psychological distress on Lack of life control showed prominent differences (0.20 vs. 0.66) in model 1. A similar pattern but less pronounced was noted for model 2 (0.43 vs. 0.58).

##### 3.4.3.3. Indirect effects – mediations

In both models, all possible indirect effects/mediating effects were significant (complementary mediations) but dependent on the model (Table 4A). In model 1, Pain intensity is a mediating construct between Psychological distress and Interference (indirect specific effect = 0.18) and between Psychological distress and Lack of life control (indirect specific effect = 0.06). The other indirect/mediating effects in model 1 were significant but small (i.e., indirect specific effects <0.05). In model 2, Psychological distress was a mediating construct between Pain intensity and Interference (indirect specific effect = 0.11) and between Pain intensity and Lack of life control (indirect specific effect = 0.22). The other indirect/mediating effects in model 2 were significant but small (i.e., indirect specific effects <0.05).

##### 3.4.3.4. Explanatory and predictive powers

In both models, the coefficient of determination (*R*^2^) was higher for Lack of life control (*R*^2 ^= 0.48) and Interference (*R*^2 ^= 0.45) than for Pain intensity (model 1)/Psychological distress (model 2) (*R*^2 ^= 0.16). The Predictive power analyses indicated that the two models had predictive relevance (Supplementary Table S5).

### 3.5. PLS-SEM in two subgroups with low and high psychological distress

#### 3.5.1. Descriptive data for two subgroups

To investigate whether psychological distress was a moderating factor, we divided the total cohort into two subgroups. One subgroup, labelled High distress subgroup, had signs of definite depression and/or anxiety according to subscales of HAD (i.e., ≥11). The other subgroup, labelled Low distress subgroup, had lower signs of depression and anxiety according to HAD (i.e., <11). Descriptive data for all variables of both subgroups are shown in Table 3. All variables except sex/gender proportions differed significantly between the two subgroups. The High distress subgroup was born outside Europe to a larger extent and had university education to a lesser degree (Table 3). The Low distress subgroup reported a better situation on all clinical variables. The largest ESs were noted for the psychological distress variables (>0.90). The other variables had lower ESs. For example, pain intensity variables were associated with small to medium ESs.

#### 3.5.2. Evaluation of the reflective measurement (outer) models

**Figure 4:**
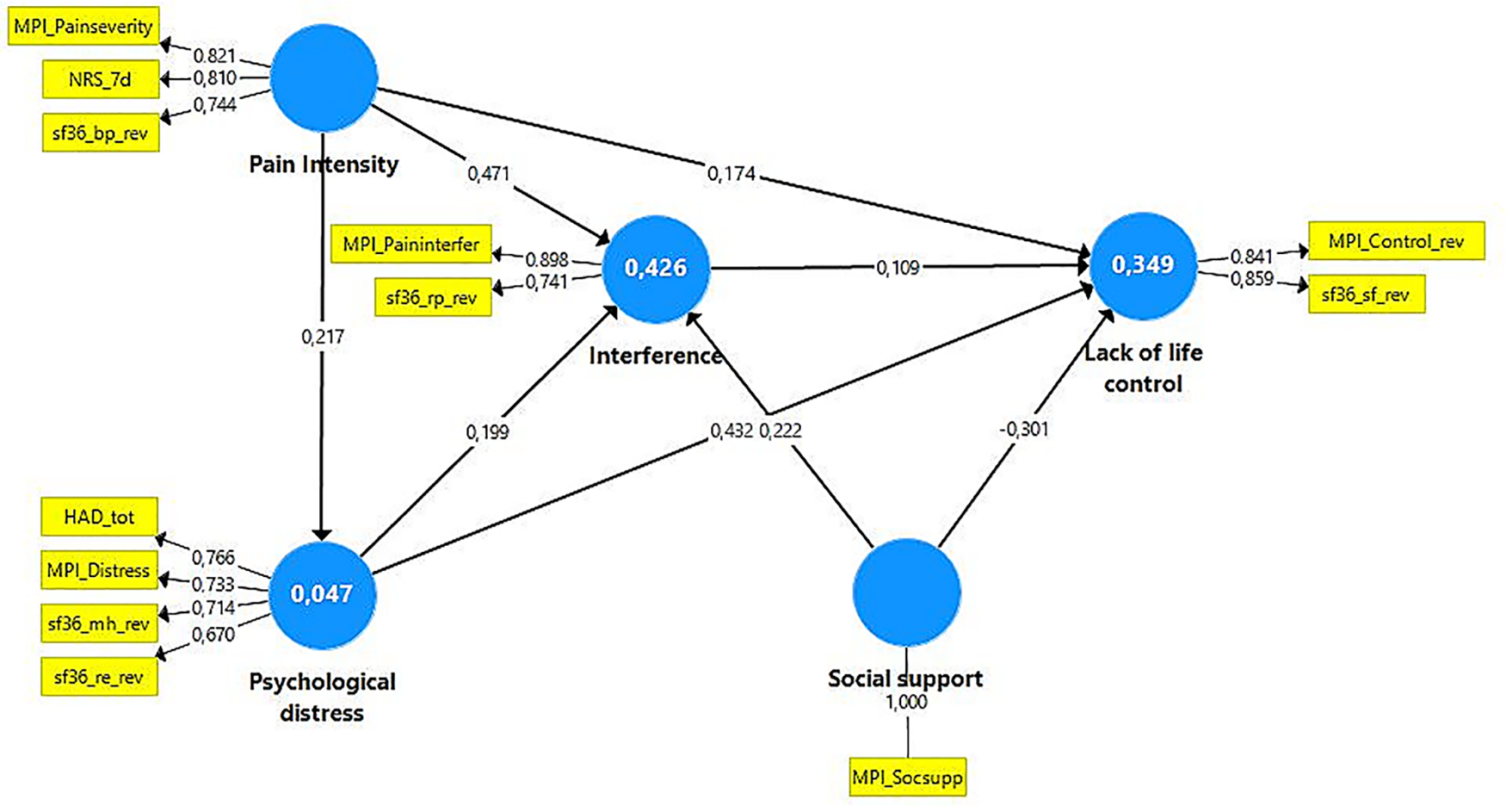
Model 2 (pain intensity affects psychological distress) for the subgroup with **low distress** (*N* = 20,986). The blue circles show the constructs. The yellow boxes show the indicators (reflective) for each latent variable; the figure attached to the thin arrows show the loading. The thicker arrows show the suggested relations between the constructs. The figure attached shows the path coefficient (direct effect). The explained variance (*R*^2^) is reported within the relevant constructs. Note that some of the MPI variables and all sf36 variables were revised (indicated with _rev in the variable name) so that high values indicated a troublesome/negative situation. NRS_7d, Pain intensity according to a numeric rating scale; HAD, The Hospital Anxiety and Depression Scale; HAD-tot, sum of the two subscales of HAD; MPI, Multidimensional Pain Inventory; MPI_Paininterfer, MPI Pain interference; MPI_Painseverity, MPI Pain severity; MPI_Socsupp, MPI Social support; sf36, The Short Form Health Survey; sf36_rp, sf36-role physical; sf36_bp, sf36-bodily pain; sf36_sf, sf36-social function; sf36_re, sf36-role emotional; sf36_mh, sf36-mental health.

**Figure 5:**
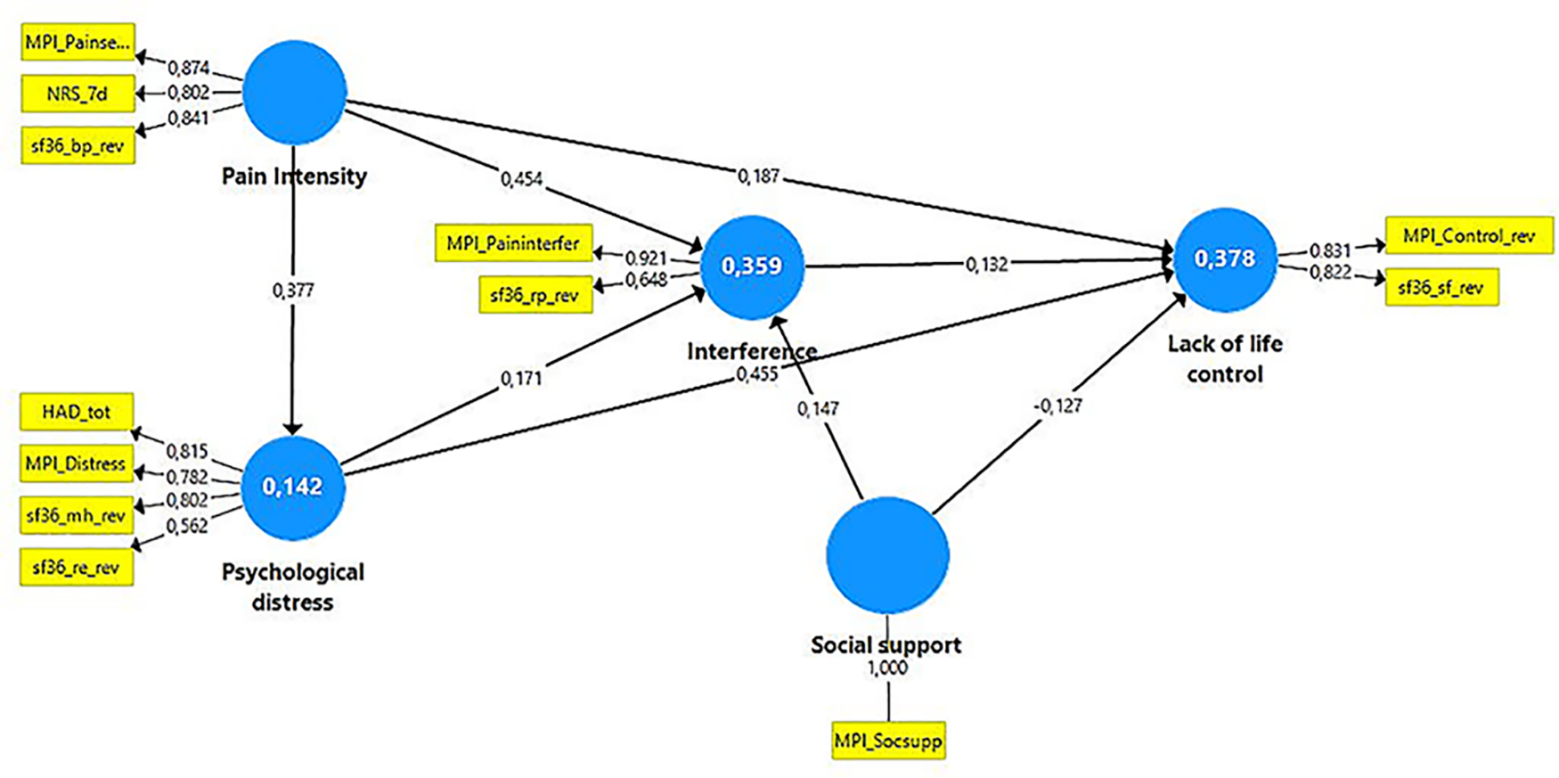
Model 2 (pain intensity affects psychological distress) for the subgroup with **high distress** (*N* = 19,288). The blue circles show the constructs. The yellow boxes show the indicators (reflective) for each latent variable; the figure attached to the thin arrows show the loading. The thicker arrows show the suggested relations between the constructs. The figure attached shows the path coefficient (direct effect). The explained variance (*R*^2^) is reported within the relevant constructs. Note that some of the MPI variables and all sf36 variables were revised (indicated with _rev in the variable name) so that high values indicated a troublesome/negative situation. NRS_7d, Pain intensity according to a numeric rating scale; HAD, The Hospital Anxiety and Depression Scale; HAD-tot, sum of the two subscales of HAD; MPI, Multidimensional Pain Inventory; MPI_Paininterfer, MPI Pain interference; MPI_Painseverity, MPI Pain severity; MPI_Socsupp, MPI Social support; sf36, The Short Form Health Survey; sf36_rp, sf36-role physical; sf36_bp, sf36-bodily pain; sf36_sf, sf36-social function; sf36_re, sf36-role emotional; sf36_mh, sf36-mental health.

Separate PLS-SEM analysis were conducted for the two subgroups using the same latent variables, the same indicators, and the same causal patterns as for model 2 of the total cohort (Figures 4, 5). The outer model of both subgroups was associated with good indicator reliability, internal consistency reliability, convergent validity, and discriminant validity. The outer model characteristics are shown in Supplementary Table S4. Indicator loadings for each construct/latent variable are shown in Figures 4, 5. Indicators with loadings >0.40 and <0.708 were not omitted since it did not result in increased internal consistency or convergent validity.

#### 3.5.3. Evaluation of the structural (inner) models

Collinearity was not an issue in the two subgroups. VIF values were below 2 in both subgroups (Low distress subgroup: 1.00–1.74; High distress subgroup: 1.00–1.60).

The direct effects (path coefficients) (Figures 4, 5), total effects, and indirect effects were all significant (all *P* < 0.001) in the two subgroups (Table 6).

**Table 6:** Path coefficients, total effects, indirect, and specific indirect effects for the Low distress (*N* = 20,986) and high distress (*N* = 19,288) subgroups. Mean ± SD, *t*-values, *P*-values, and 95% confidence intervals (95% CI) are reported.

Path coefficients	Low distress sub-group				High distress sub-group				Sign
	Mean ± SD	*t*	*P*	95% CI	Mean SD	*t*	*P*	95% CI	
Pain intensity → Psychological distress	0.217 ± 0.008	26.866	<0.001	0.201–0.233	0.377 ± 0.006	58.047	<0.001	0.365–0.390	Y
Pain intensity → Interference	0.471 ± 0.006	82.301	<0.001	0.460–0.482	0.454 ± 0.008	55.983	<0.001	0.438–0.470	N
Pain intensity → Lack of life control	0.174 ± 0.009	18.926	<0.001	0.156–0.192	0.187 ± 0.008	22.244	<0.001	0.170–0.203	N
Psychological distress → Interference	0.199 ± 0.006	33.917	<0.001	0.188–0.211	0.171 ± 0.007	25.221	<0.001	0.158–0.185	Y
Psychological distress → Lack of life control	0.432 ± 0.006	73.598	<0.001	0.421–0.444	0.455 ± 0.006	72.266	<0.001	0.442–0.467	Y
Social support → Interference	0.222 ± 0.006	34.145	<0.001	0.209–0.235	0.147 ± 0.007	21.61	<0.001	0.134–0.161	Y
Social support → Lack of life control	−0.301 ± 0.007	44.287	<0.001	−0.314 to −0.287	−0.127 ± 0.006	20.59	<0.001	−0.139 to −0.115	Y
Interference → Lack of life control	0.109 ± 0.008	13.418	<0.001	0.093–0.125	0.132 ± 0.009	15.174	<0.001	0.115–0.148	N
**Total effects**									
Pain intensity → Psychological distress	0.217 ± 0.008	26.654	<0.001	0.201–0.233	0.377 ± 0.006	58.047	<0.001	0.365–0.390	Y
Pain intensity → Interference	0.514 ± 0.005	95.654	<0.001	0.504–0.525	0.519 ± 0.007	74.31	<0.001	0.505–0.532	N
Pain intensity → Lack of life control	0.324 ± 0.010	33.13	<0.001	0.305–0.342	0.427 ± 0.007	58.877	<0.001	0.412–0.441	Y
Psychological distress → Interference	0.199 ± 0.006	33.541	<0.001	0.188–0.211	0.171 ± 0.007	25.221	<0.001	0.158–0.185	Y
Psychological distress → Lack of life control	0.454 ± 0.006	82.178	<0.001	0.443–0.465	0.477 ± 0.006	79.131	<0.001	0.466–0.489	Y
Social support → Interference	0.222 ± 0.007	33.955	<0.001	0.209–0.235	0.147 ± 0.007	21.61	<0.001	0.134–0.161	Y
Social support → Lack of life control	−0.276 ± 0.007	39.739	<0.001	−0.29 to 0.263	−0.107 ± 0.006	17.074	<0.001	−0.120 to −0.095	Y
Interference → Lack of life control	0.109 ± 0.008	13.475	<0.001	0.093–0.125	0.132 ± 0.009	15.174	<0.001	0.115–0.148	N
**Indirect effects**									
Pain intensity → Psychological distress	NA	NA	NA	NA	NA	NA	NA	NA	NA
Pain intensity → Interference	0.043 ± 0.002	19.472	<0.001	0.039–0.048	0.065 ± 0.003	22.652	<0.001	0.059–0.070	Y
Pain intensity → Lack of life control	0.150 ± 0.005	28.552	<0.001	0.140–0.160	0.240 ± 0.005	44.292	<0.001	0.229–0.251	Y
Psychological distress → Interference	NA	NA	NA	NA	NA	NA	NA	NA	NA
Psychological distress → Lack of life control	0.022 ± 0.002	12.263	<0.001	0.018–0.025	0.023 ± 0.002	12.832	<0.001	0.019–0.026	N
Social support → Interference	NA	NA	NA	NA	NA	NA	NA	NA	NA
Social support → Lack of life control	0.024 ± 0.002	12.816	<0.001	0.020–0.028	0.019 ± 0.001	13.327	<0.001	0.017–0.022	N
Interference → Lack of life control	NA	NA	NA	NA	NA	NA	NA	NA	NA
**Specific indirect effects**									
Pain intensity → Psychological distress → Interference	0.043 ± 0.002	19.472	<0.001	0.039–0.048	0.065 ± 0.003	22.652	<0.001	0.059–0.070	Y
Pain intensity → Interference → Lack of life control	0.051 ± 0.004	13.231	<0.001	0.044–0.059	0.060 ± 0.004	14.568	<0.001	0.052–0.068	N
Pain intensity → Psychological distress → Lack of life control	0.094 ± 0.004	26.757	<0.001	0.087–0.101	0.172 ± 0.004	45.106	<0.001	0.164–0.179	Y
Pain intensity → Psychological distress → Interference → Lack of life control	0.005 ± 0.000	10.847	<0.001	0.004–0.006	0.009 ± 0.001	12.497	<0.001	0.007–0.010	Y
Psychological distress → Interference → Lack of life control	0.022 ± 0.002	12.263	<0.001	0.018–0.025	0.023 ± 0.002	12.832	<0.001	0.019–0.026	N
Social support → Interference → Lack of life control	0.024 ± 0.002	12.816	<0.001	0.02–0.028	0.019 ± 0.001	13.327	<0.001	0.017–0.022	N

N, not significant (i.e., overlapping 95% CI); NA, not applicable; Y, significant (i.e., non-overlapping 95% CI for the two subgroups).

##### 3.5.3.1. Direct effects (path coefficients)

Scrutinizing the 95% CIs for the coefficients pairwise between the two subgroups, we found some obvious significant differences in directs effects (path coefficients) between the two subgroups (Table 6, Figures 4, 5). The direct effect (path coefficient) between Pain intensity and Psychological distress was significantly stronger in the High distress group than in the Low distress group; 0.38 (medium *f*^2^) vs. 0.22 (small *f*^2^). Pain intensity vs. Interference had relatively high direct effects in both subgroups but did not differ significantly between the two subgroups (0.47 vs. 0.45; both medium *f*^2^). No significant differences were noted for the direct effects of Pain intensity vs. Lack of life control between the High distress subgroup and Low distress subgroup (0.19 vs. 0.17; both small *f*^2^). Psychological distress vs. Interference had a somewhat stronger direct effect in the Low distress group than in the High distress group (0.20 vs. 0.17; both small *f*^2^). Relatively high direct effects were noted for the Psychological distress - Lack of life control relationship in the Low distress and High distress subgroups (0.43 vs. 0.46; both medium *f*^2^). Stronger direct effects in the Low distress subgroup than in the High distress subgroup were noted for the relations between Social support and Interference and Lack of life control, respectively (all groups with small *f*^2^). No significant subgroup difference existed in the direct effects for Interference vs. Lack of life control (Low distress: 0.11 vs. High distress: 0.13; both with no measurable effect).

##### 3.5.3.2. Total effects

The total effect of Pain intensity vs. Interference did not differ significantly between the two subgroups (Table 6). The total effect of Pain intensity on Lack of life control differed significantly between the two subgroups and was strongest in the High distress subgroup (0.43 vs. 0.32). The total effect for Psychological distress vs. Interference was significantly weaker in the High distress group than the Low distress group (0.17 vs. 0.20). In the High distress subgroup, the total effect for the Psychological distress-Lack of life control relationship was somewhat (significantly) higher than in the Low distress subgroup (0.48 vs. 0.45) (Table 6). Social support had weaker absolute total effects in the High distress subgroup than in the Low distress group (Social support-Interference relation: 0.15 vs. 0.22; Social support-Lack of life control relation: −0.11 vs. −0.28).

##### 3.5.3.3. Indirect effects – mediations

In the Low distress group, Psychological distress was a mediating construct between Pain intensity and Lack of life control (indirect specific effect = 0.09). Furthermore, Interference was a mediating construct between pain Intensity and Lack of life control (indirect specific effect = 0.05). In the High distress subgroup, Psychological distress was a mediating construct between Pain intensity and Lack of life control (indirect specific effect = 0.17) and between Pain intensity and Interference (indirect specific effect = 0.07). In addition, Interference was a mediating construct between Pain intensity and Lack of life control (indirect specific effect = 0.06). Hence, the mediating effects were stronger for these three relationships in the High distress subgroup.

##### 3.5.3.4. Explanatory powers and predictive powers

The coefficients of determination *R*^2^ for Psychological distress and Interference differed significantly between the two subgroups (Figures 4, 5). Hence, *R*^2^ of psychological distress was lower in the Low distress group (*R*^2 ^= 0.047 ± 0.004, *P* < 0.001, 95% CI: 0.041–0.054) than in the High distress group (*R*^2 ^= 0.142 ± 0.005 (*P* < 0.001, 95% CI: 0.133–0.152). A significantly higher *R*^2^ for Interference was found in the Low distress subgroup (*R*^2 ^= 0.426 ± 0.006, *P* < 0.001, 95% CI: 0.415–0.437) compared to the High distress group (*R*^2 ^= 0.359 ± 0.007, *P* < 0.001, 95% CI: 0.345–0.373). The explained variances for Lack of life control did not differ significantly between the low distress group (*R*^2 ^= 0.349 ± 0.006, *P* < 0.001, 95% CI: 0.337–0.360) and the high distress group (*R*^2 ^= 0.378 ± 0.006, *P* < 0.001, 95%CI: 0336–0.391). *Q*^2^_predictive_ values indicated that the models of the two subgroups had predictive relevance except for sf36-mh-rev in the Low distress subgroup (Supplementary Table S5).

## 4. Discussion

### 4.1. Major results

Modern clinical pain management relies on the biopsychosocial model of pain. The model is usually presented graphically in a relatively simple way (74–76). However, associated explanations of the model suggest significant complexity between the constituents of the three subcomponents. But these relationships are only partially known. PLS-SEM can contribute to the understanding of these relationships. Hence, this real-life study of more than 40,000 patients investigated to what extent and how Pain intensity, Psychological distress, and Social support interact with Interference and Lack of life control aspects.

Although high prevalence of psychological distress was found in the cohort, relatively low correlation and explanatory power existed for the Pain intensity - Psychological distress relationship. Pain intensity had stronger effect on Interference than on Lack of life control, whereas Psychological distress had stronger effect on Lack of life control than on Interference. Depending on the underlying assumption concerning the Pain intensity - Psychological distress relationship, Pain intensity and Psychological distress could complementarily mediate each other in relation to the two impact constructs (Interference and Lack of life control). Social support showed very similar absolute correlations with Interference and Lack of life control. Interference and Lack of life control showed relatively weak associations. Psychological distress level was a moderating factor for several of the significant and important associations/paths.

### 4.2. Pain intensity-psychological distress relationships

Chronic pain conditions are associated with high prevalence of anxiety and depressive symptoms (77–80). This does not necessarily mean that high correlations exist between pain intensity and psychological distress levels. The present and other studies have shown that the intercorrelations between pain intensity and psychological distress levels are relatively low (24, 81, 82) – 16% of the variance explained (*R*^2^) in this study. As noted above, pain intensity and psychological distress are intercorrelated both in cross sectional and longitudinal studies. Also, day-to-day pain variability studies generally indicate reciprocal associations with negative affect factors (83, 84). Closely related to day-to-day fluctuations are the concepts of trait and state, which are relevant both for pain intensity and psychological distress (85–87). Several models including pain and psychological distress relationships (e.g., the misdirected problem-solving model and the fear avoidance model) suggest mediating factors between these two constructs (7, 88, 89). Cohort heterogeneity for the relationships between pain and mood aspects further complicates the understanding of pain intensity and psychological distress interactions both in short- and long-term perspectives (91, 94). Future studies of mediating (transdiagnostic) factors (e.g., attention, cognition, coping strategies, and emotion regulation) for pain intensity and psychological distress may shed further light on their intricate relationship even though understanding causal directions may be problematic.

### 4.3. Pain intensity and psychological distress vs. Interference and Lack of life control

As expected in the literature, we observed that both Pain intensity and Psychological distress were positively and significantly associated with the Interference and Lack of life control constructs (24, 81) (Figure 3). Relatively similar explanatory power (*R*^2^) was found for Interference and Lack of life control (0.45 vs. 0.48) in the total cohort (Figure 3). Thus, also other factors than Pain intensity, Psychological distress, and Social support contribute to the explanatory power of these constructs.

For both models of the total cohort, Pain intensity had stronger total effects on Interference than on Lack of life control, whereas the reverse was found for Psychological distress vs. Interference and Lack of life control (Table 4A). These results are consistent with results from another study of this cohort using other methods. The study found that pain intensity variables were more important for interference than for depressive symptoms, whereas life control was more strongly associated with symptoms of anxiety and depression (38). In our network study, anxiety and depression levels had stronger relationships than pain intensity with life control aspects (24).

The relative importance of Pain intensity and Psychological distress for the two impact constructs depend on the underlying assumption concerning their interrelationship. Hence, model differences were also obvious. For instance, the total effects of Pain intensity vs. both Interference and Lack of life control were markedly larger in model 2 than in model 1 (Table 4A). Psychological distress had relatively weaker total effects on Interference in model 2 than in model 1 (Table 4A). Differences also existed across the two models when comparing the relative contributions of Pain intensity and Psychological distress for the explanation of Interference and Lack of life control. For example, Pain intensity and Psychological distress in model 1 had very similar total effects on Interference, whereas Pain intensity in model 2 showed a significantly stronger total effect than psychological distress on Interference (0.56 vs. 0.26). In addition, the indirect (mediating) effects were significant and model dependent (Tables 4A,B). Psychological stress and Pain intensity can complementarily mediate each other in relation to Interference and Lack of life control (Tables 4A,B).

The reports of heterogeneity for the causal directions between pain intensity and psychological distress must reasonably have clinical implications (91–94). Thus, the clinical management must take this heterogeneity into account when designing individual treatment interventions.

### 4.4. Interference and Lack of life control relationship

Interference and Lack of life control showed relatively weak positive correlations, which suggests that they are different constructs of the umbrella construct of life impact. Clinically, they should be assessed separately. Although several large studies from the SQRP, including this one, have focused on factors that intercorrelate with these two separate constructs of life impact, more studies from large real-life cohorts that can identify variables with significant associations with interference and lack of life control are desirable (12, 24, 38). One possible limitation is that we chose indicators from different instruments – e.g., MPI and sf36 – to cover the two constructs. However, the instruments and their subscales are well established in pain research.

### 4.5. Social support showed very similar correlations with Interference and Lack of life control

Chronic pain increases the risk for social isolation, whereas social support can improve coping, management of treatments, and faster recovery of the pain condition (40, 95). Ferreira-Valente et al. reported negative associations between social support aspects and pain interference (48). A cross-sectional analysis of social isolation, which can be viewed as a negative aspect of social support, showed significant correlations both with pain interference (positive association) and physical function (negative association) (49). As in these studies, we noted that Social support was associated with decreased levels of Lack of life control (i.e., a negative association). Paradoxically, Social support correlated positively with Interference. We have no definite explanation for this counterintuitive finding. It could be due to lack of a mediating factor in our theoretical model. Hence, a recent study investigated social isolation in relation to physical function and identified depression as a mediating factor (49).

### 4.6. Psychological distress level as a moderator

The presence or non-presence of severe psychological distress according to HAD was a moderating factor. Thus, several intercorrelations between the constructs/latent variables depend on whether definite signs of anxiety and/or depression are present. Specifically, Pain intensity, Psychological distress, and Social support affect Interference and Lack of life control with different strengths in the two subgroups. For example, Pain intensity and Psychological distress were more strongly intercorrelated in the High distress subgroup than in the Low distress subgroup (Figures 4, 5). Moreover, Psychological distress showed significantly stronger direct effects on Lack of life control but weaker direct effects on Interference in the High distress subgroup compared to the Low distress subgroup. In addition, Interference was associated with lower coefficients of determination (*R*^2^) in the High distress subgroup.

Such heterogeneity was also noted for the effects of Social support on Interference and Lack of life control (Figures 4, 5). Hence, the buffering effect of Social support is different and, in some aspects, diminished in the most severe chronic pain conditions, which might be interpreted as social support being of greater value for the ability to be physically active and lead meaningful social lives in chronic patients with a lesser degree of psychological co-morbidity. We also found that the three most important complementary mediating effects were stronger in the High distress subgroup.

Our results taken together indicate cohort heterogeneity and emphasise the need to subgroup patients with complex chronic pain conditions. In the literature there are several studies identifying subgroups [for references see (12)]. There is no agreement concerning the important input variables for such subgroupings; most studies have been hypothesis driven. This study as well as other studies indicate that psychological variables are important (96–101). There is a need for data driven methods in large cohorts of chronic pain patients for identifying the relevant input variables (12, 38). Studies using such methods also identify pain intensity aspects as important for the heterogeneity. Furthermore, studies including from SQRP have found that subgroups show different outcomes of IPRP (12, 51, 53). The different treatment outcomes could partially be due to the presence of moderating effects from definite anxiety and/or depression as well as from other currently unknown variables. Future research needs to focus more on identifying moderating factors. Covering such a knowledge gap may be important for improving outcomes of rehabilitation interventions.

### 4.7. Strengths and limitations

A strength is the large number of chronic pain patients with nation-wide representation. Our results are relevant for patients referred to specialist care. Women are overrepresented in specialist clinics in Sweden compared to the community prevalence (12, 96). In an earlier large SQRP study we could not confirm reports that women assessed at specialist clinics have a more severe clinical situation than men or are judged to be more prone to behavioral change (50, 102). The reasons for this skewed assortment/selection are unclear and need to be addressed. All constructs except Social support had several indicators, which were scales of well-established instruments. This is an advantage from a measurement error point of view compared to path analyses, which only use single items or scales representing one aspect/construct. A limitation is that our theoretical model and subsequent analyses is based on cross-sectional data. For example, it was not possible to determine the most common causal relationship between pain intensity and psychological distress, which is a disadvantage of our models. Our construct Interference covers pain interference and physical function. These aspects are often used interchangeably, but recent reports suggest that it may be necessary to differentiate these measures in, for example, longitudinal perspectives (47, 103).

## 5. Conclusions

The results are important both for the assessments and the design of treatments for patients with chronic pain. In this large real-life study of more than 40,000 patients, a relatively low correlation for the Pain intensity - Psychological distress relationship was found. A clinical treatment consequence may be that lowering one of them may not result in lowering the other. Similarly, Interference and Lack of life control showed relatively weak associations, which emphasises the need to clinically assess them separately. Pain intensity had a stronger effect on Interference than on Lack of life control, whereas Psychological distress had a stronger effect on Lack of life control than on Interference. Thus, it seems that anxiety and depression impair function and the ability to lead socially meaningful lives to a greater extent, whereas higher pain levels have a more detrimental effect on physical functioning in chronic pain states. The relative strengths of Pain intensity and Psychological distress on the two impact constructs depend on the underlying assumption concerning the Pain intensity-Psychological distress relationship. Social support influenced both impact constructs investigated although to a greater extent in individuals with chronic pain who had lower clinical severity – i.e., a definite sign (or lack of sign) of anxiety and/or depression was a moderating factor.

## Data Availability

The raw data supporting the conclusions of this article will be made available by the authors, without undue reservation.

## Footnotes

^1^Throughout the text, we capitalize the initial letter of constructs/latent variables – e.g., Interference and Lack of life control.
