# Supplementary material for: Pain intensity and psychological distress show different associations with interference and lack of life control: A clinical registry-based cohort study of >40,000 chronic pain patients from SQRP
Source: Front Pain Res (Lausanne). 2023 Mar 2;4:1093002. doi: 10.3389/fpain.2023.1093002 (PMC10017552; doi:10.3389/fpain.2023.1093002)
Supplement: Supplementary file 1 [file Table1.docx]

Supplementary Material

# Supplementary Digital Content Table 1: OPLS regressions of four psychological distress variables. Variables in bold type are significant (i.e., VIP≥1.0).

| ***HAD-tot*** | ***OPLS*** |  | ***MPI-Distress*** | ***OPLS*** |  | ***sf36-mental health*** | ***OPLS*** |  | ***sf36-role emotional*** | ***OPLS*** |  |
| --- | --- | --- | --- | --- | --- | --- | --- | --- | --- | --- | --- |
| Variable | VIPpred | p(corr) | Variable | VIPpred | p(corr) | Variable | VIPpred | p(corr) | Variable | VIPpred | p(corr) |
| **MPI-Control** | **1.79** | **-0.73** | **MPI-Pain interference** | **1.83** | **0.77** | **MPI-Control** | **2.15** | **0.83** | **sf36-social function** | **2.00** | **0.82** |
| **sf36-social function** | **1.74** | **-0.71** | **MPI-Pain severity** | **1.71** | **0.72** | **sf36-social function** | **2.14** | **0.83** | **MPI-Control** | **1.81** | **0.74** |
| **MPI-Pain interference** | **1.59** | **0.65** | **MPI-Control** | **1.41** | **-0.60** | **sf36-bodily pain** | **1.37** | **0.53** | **sf36-role physical** | **1.51** | **0.62** |
| **MPI-Pain severity** | **1.26** | **0.51** | **NRS-7d** | **1.34** | **0.57** | **sf36-physical function** | **1.27** | **0.49** | **sf36-bodily pain** | **1.23** | **0.50** |
| **NRS-7d** | **1.08** | **0.44** | **sf36-social function** | **1.30** | **-0.55** | Outside-Europe | 0.94 | -0.36 | **MPI-Pain interference** | **1.16** | **-0.47** |
| **sf36-bodily pain** | **1.02** | **-0.42** | **sf36-bodily pain** | **1.05** | **-0.45** | MPI-Pain interference | 0.63 | -0.24 | sf36-physical function | 0.96 | 0.39 |
| PRI | 0.91 | 0.37 | PRI | 0.80 | 0.34 | sf36-role physical | 0.62 | 0.24 | Outside-Europe | 0.74 | -0.30 |
| sf36-role physical | 0.74 | -0.30 | sf36-role physical | 0.72 | -0.30 | MPI-Social support | 0.53 | 0.21 | MPI-Pain severity | 0.66 | -0.27 |
| Outside-Europe | 0.71 | 0.29 | MPI-Social support | 0.59 | 0.25 | NRS-7d | 0.43 | -0.17 | NRS-7d | 0.64 | -0.26 |
| sf36-physical function | 0.67 | -0.27 | Age | 0.55 | -0.23 | MPI-Pain severity | 0.26 | -0.10 | PRI | 0.39 | -0.16 |
| University | 0.29 | -0.12 | Outside-Europe | 0.37 | 0.16 | PRI | 0.25 | -0.10 | University | 0.29 | 0.12 |
| Age | 0.24 | -0.10 | sf36-physical function | 0.34 | -0.15 | Age | 0.17 | 0.06 | Gender | 0.19 | -0.08 |
| MPI-Social support | 0.09 | 0.04 | University | 0.29 | -0.12 | University | 0.16 | 0.06 | MPI-Social support | 0.11 | 0.05 |
| Gender | 0.07 | 0.03 | Pain-duration | 0.12 | -0.05 | Gender | 0.15 | -0.06 | Pain-duration | 0.07 | 0.03 |
| Pain-duration | 0.06 | 0.02 | Gender | 0.01 | -0.01 | Pain-duration | 0.08 | 0.03 | Age | 0.03 | 0.01 |
| R^2^ | 0.44 |  | R^2^ | 0.48 |  | R^2^ | 0.48 |  | R^2^ | 0.25 |  |
| Q^2^ | 0.45 |  | Q^2^ | 0.48 |  | Q^2^ | 0.48 |  | Q^2^ | 0.25 |  |
| CV-ANOVA *P* | <0.001 |  | CV-ANOVA *P* | <0.001 |  | CV-ANOVA *P* | <0.001 |  | CV-ANOVA *P* | <0.001 |  |
| N | 40 184 |  | N | 40 184 |  | N | 40 184 |  | N | 40 184 |  |

NRS-7d= Pain intensity according to a numeric rating scale; University= University education (binary variable); Outside-Europe=born outside Europe (binary variable), PRI= Pain region Index; HAD= The Hospital Anxiety and Depression Scale; HAD-tot= sum of the two subscales of HAD; MPI=Multidimensional Pain Inventory; sf36= The Short Form Health Survey
