# Supplementary material for: Pain intensity and psychological distress show different associations with interference and lack of life control: A clinical registry-based cohort study of >40,000 chronic pain patients from SQRP
Source: Front Pain Res (Lausanne). 2023 Mar 2;4:1093002. doi: 10.3389/fpain.2023.1093002 (PMC10017552; doi:10.3389/fpain.2023.1093002)
Supplement: Supplementary file 2 [file Table2.docx]

Supplementary Material

# Supplementary Digital Content Table 2: OPLS regressions of the three pain intensity variables. Variables in bold type are significant (i.e., VIP≥1.0).

| ***NRS-7D*** | ***OPLS*** |  | ***MPI-Pain Severity*** | ***OPLS*** |  | ***sf36-bodily pain*** | ***OPLS*** |  |
| --- | --- | --- | --- | --- | --- | --- | --- | --- |
| Variable | VIPpred | p(corr) | Variable | VIPpred | p(corr) | Variable | VIPpred | p(corr) |
| **MPI-Pain interference** | **2.02** | **0.86** | **MPI-Pain interference** | **2.33** | **0.91** | **sf36-physical function** | **1.62** | **0.76** |
| **MPI-Distress** | **1.60** | **0.68** | **MPI-Distress** | **1.73** | **0.67** | **sf36-social function** | **1.54** | **0.72** |
| **HAD-tot** | **1.29** | **0.55** | **MPI-Social support** | **1.46** | **0.57** | **sf36-role physical** | **1.32** | **0.62** |
| **MPI-Social support** | **1.17** | **0.50** | **HAD-tot** | **1.21** | **0.47** | **MPI-Pain interference** | **1.31** | **-0.61** |
| **sf36-physical function** | **1.02** | **-0.43** | sf36-role physical | 0.71 | -0.28 | **MPI-Control** | **1.19** | **0.56** |
| sf36-social function | 0.83 | -0.35 | sf36-physical function | 0.61 | -0.24 | **sf36-mental health** | **1.07** | **0.50** |
| sf36-role physical | 0.78 | -0.33 | sf36-social function | 0.60 | -0.23 | HAD-tot | 0.96 | -0.45 |
| MPI-Control | 0.77 | -0.33 | University | 0.48 | -0.19 | MPI-Distress | 0.92 | -0.43 |
| University | 0.65 | -0.28 | sf36-role emotional | 0.41 | -0.16 | sf36-role emotional | 0.83 | 0.39 |
| sf36-role emotional | 0.56 | -0.24 | MPI-Control | 0.39 | -0.15 | MPI-Social support | 0.52 | -0.25 |
| sf36-mental health | 0.55 | -0.24 | sf36-mental health | 0.26 | -0.10 | Outside-Europe | 0.45 | -0.21 |
| Outside-Europe | 0.50 | 0.21 | Gender | 0.25 | -0.10 | University | 0.26 | 0.12 |
| Gender | 0.29 | -0.12 | Outside-Europe | 0.21 | 0.08 | Gender | 0.11 | 0.05 |
| Age | 0.10 | 0.04 | Age | 0.01 | 0.00 | Age | 0.11 | -0.05 |
| R^2^ | 0.29 |  | R^2^ | 0.57 |  | R^2^ | 0.43 |  |
| Q^2^ | 0.28 |  | Q^2^ | 0.57 |  | Q^2^ | 0.43 |  |
| CV-ANOVA *P* | <0.001 |  | CV-ANOVA *P* | <0.001 |  | CV-ANOVA *P* | <0.001 |  |
| N | 40 184 |  | N | 40 184 |  | N | 40 184 |  |

NRS-7d= Pain intensity according to a numeric rating scale; University= University education (binary variable); Outside-Europe=born outside Europe (binary variable), PRI= Pain region Index; HAD= The Hospital Anxiety and Depression Scale; HAD-tot= sum of the two subscales of HAD; MPI=Multidimensional Pain Inventory; sf36= The Short Form Health Survey
