# Supplementary material for: Pain intensity and psychological distress show different associations with interference and lack of life control: A clinical registry-based cohort study of >40,000 chronic pain patients from SQRP
Source: Front Pain Res (Lausanne). 2023 Mar 2;4:1093002. doi: 10.3389/fpain.2023.1093002 (PMC10017552; doi:10.3389/fpain.2023.1093002)
Supplement: Supplementary file 3 [file Table3.docx]

Supplementary Material

# Supplementary Digital Content Table 3: Outer model characteristics of the two models using all subjects. All these characteristics were identical in the two models. In model 1, psychological distress affects pain. In model 2, pain affects psychological distress.

|  | ***Model 1 & 2*** |
| --- | --- |
| ***Internal consistency reliability***  Exact (composite) reliability coefficient (ρ_A_)  Limit: >0.50 |  |
| *Psychological distress* | 0.89 |
| *Pain intensity* | 0.87 |
| *Interference* | 0.81 |
| *Social support* | 1.00 |
| *Lack of life control* | 0.86 |
| ***Convergent validity***  Average variance extracted (AVE) Limit: >0.50 |  |
| *Psychological distress* | 0.67 |
| *Pain intensity* | 0.68 |
| *Interference* | 0.68 |
| *Social support* | 1.00 |
| *Lack of life control* | 0.75 |
| ***Discriminant validity***  Heterotrait-monotrait ratio (HTMT)  Lower than 0.90? Yes (Y)/No(N) |  |
| *Psychological distress* | Y |
| *Pain intensity* | Y |
| *Interference* | Y |
| *Social support* | Y |
| *Lack of life control* | y |
