# Supplementary material for: Pain intensity and psychological distress show different associations with interference and lack of life control: A clinical registry-based cohort study of >40,000 chronic pain patients from SQRP
Source: Front Pain Res (Lausanne). 2023 Mar 2;4:1093002. doi: 10.3389/fpain.2023.1093002 (PMC10017552; doi:10.3389/fpain.2023.1093002)
Supplement: Supplementary file 4 [file Table4.docx]

Supplementary Material

# Supplementary Digital content Table 4: Outer model characteristics for the two subgroups Low distress (N=20 986) and High distress (N=19 288). Loadings are shown in Figures 3 and 4.

| ***Latent variables*** | ***Low Distress subgroup*** | ***High Distress subgroup*** |
| --- | --- | --- |
| ***Internal consistency reliability*** Exact (composite) reliability coefficient (ρ_A_) Limit: >0.50 |  |  |
| *Psychological distress* | 0.81 | 0.83 |
| *Pain intensity* | 0.84 | 0.88 |
| *Interference* | 0.81 | 0.77 |
| *Social support* | 1.00 | 1.00 |
| *Lack of life control* | 0.84 | 0.81 |
| ***Convergent validity*** Average variance extracted (AVE)  Limit: >0.50 |  |  |
| *Psychological distress* | 0.52 | 0.56 |
| *Pain intensity* | 0.63 | 0.71 |
| *Interference* | 0.68 | 0.63 |
| *Social support* | 1.00 | 1.00 |
| *Lack of life control* | 0.72 | 0.68 |
| ***Discriminant validity*** Heterotrait-monotrait ratio (HTMT) Lower than 0.90? Yes (Y)/No (N) |  |  |
| *Psychological distress* | Y | Y |
| *Pain intensity* | Y except vs. Interference (0.91) | Y |
| *Interference* | Y except vs. Pain intensity (0.91) | Y |
| *Social support* | Y | Y |
| *Lack of life control* | Y | Y |
