# Supplementary material for: Pain intensity and psychological distress show different associations with interference and lack of life control: A clinical registry-based cohort study of >40,000 chronic pain patients from SQRP
Source: Front Pain Res (Lausanne). 2023 Mar 2;4:1093002. doi: 10.3389/fpain.2023.1093002 (PMC10017552; doi:10.3389/fpain.2023.1093002)
Supplement: Supplementary file 5 [file Table5.docx]

Supplementary Material

# Supplementary Digital content Table 5: Q^2^_predict_ results for target constructs for the two models of the total cohort and for the two subgroups.

| ***Model 1*** | ***all subjects*** | ***Model 2*** | ***all subjects*** | ***Low distress*** | ***subgroup*** | ***High distress*** | ***subgroup*** |
| --- | --- | --- | --- | --- | --- | --- | --- |
| **Q^2^_predict_** |  | **Q^2^_predict_** |  | **Q^2^_predict_** |  | **Q^2^predict** |  |
| sf36-social function-rev | 0.310 | sf36-social function-rev | 0.154 | sf36-social function-rev | 0.107 | MPI-Control-rev | 0.123 |
| MPI-Control-rev | 0.343 | MPI-Control-rev | 0.134 | MPI-Control-rev | 0.103 | sf36-social function-rev | 0.112 |
| MPI-Pain interference | 0.283 | MPI-Pain interference | 0.425 | MPI-Pain interference | 0.419 | sf36-role physical-rev | 0.045 |
| sf36-role physical-rev | 0.080 | sf36-role physical-rev | 0.080 | sf36-role physical-rev | 0.080 | MPI-Pain interference | 0.351 |
| NRS-7d | 0.080 | HAD-tot | 0.131 | sf36-mental health-rev | -0.024 | sf36-mental health-rev | 0.044 |
| MPI-Pain severity | 0.115 | sf36-role emotional-rev | 0.041 | MPI-Distress | 0.086 | MPI-Distress | 0.166 |
| sf36-bodily pain-rev | 0.121 | MPI-Distress | 0.201 | sf36-role emotional-rev | 0.008 | sf36-role emotional-rev | 0.007 |
|  |  | sf36-mh-rev | 0.041 | HAD-tot | 0.026 | HAD-tot | 0.079 |

Note that some of the MPI variables and all sf36 variables were revised (indicated with -rev in the variable name). NRS-7d= Pain intensity according to a numeric rating scale; HAD=The Hospital Anxiety and Depression Scale; HAD-tot= sum of the two subscales of HAD; MPI=Multidimensional Pain Inventory; sf36= The Short Form Health Survey
